# Extremely Rare Case of Vulvar Myxoid Epithelioid Sarcoma

**DOI:** 10.1155/2015/971217

**Published:** 2015-02-08

**Authors:** Joana Lima Rego, Georgia Fontes Cintra, Ana Karina Junqueira Netto, Lucas Faria Abrahão-Machado, Audrey Tsunoda

**Affiliations:** ^1^Gynecologic Department, Bissaya Barreto Maternity Hospital, CHUC, 3000-059 Coimbra, Portugal; ^2^Gynecologic Oncologic Department, Barretos Cancer Hospital, SP, Brazil; ^3^Radiology Department, Barretos Cancer Hospital, SP, Brazil; ^4^Pathology Department, Barretos Hospital, SP, Brazil

## Abstract

Epithelioid sarcoma is a distinct sarcoma type with specific morphology and immunophenotype. An epithelioid sarcoma of the vulva is an extremely rare and aggressive tumor and most commonly occurs on the labia majora in women of reproductive age. Only few cases have been reported, especially with the presence of focal myxoid changes. Early diagnosis is difficult because of its benign appearance as a painless subcutaneous nodule. Optimal treatment is not well established due to its rarity. We report a successfully approached case of vulvar epithelioid sarcoma that occurred in a 34-year-old female patient, treated with wide local excision, and review of the current medical literature.

## 1. Introduction

Epithelioid sarcoma (ES) is a malignant soft tissue tumor that was established as a distinct disease entity by Enzinger in 1970 [1]. Epithelioid sarcomas occurring in the perineum and pubic regions have a higher rate of local recurrence and distant metastasis than those occurring in the upper and lower extremities. ES is classified into “distal type” and “proximal type,” according to the site of occurrence, in the upper and lower extremities or in the trunk and pubic region, respectively [2]. The incidence of primary sarcoma of the vulva is about 1.5–5% of all malignant tumors of the vulva. It occurs most frequently in the labia majora, followed, in decreasing order, by the Bartholin gland, clitoris, and labium minus [3]. The natural history of ES typically entails local recurrences and a substantial potential for distant metastasis. ES is a distinct sarcoma type with specific morphology and immunophenotype. It has long been recognized that ES may display focal myxoid change as a rare appearance. The molecular pathogenesis is poorly understood. Optimal treatment for ES of the vulva remains controversial due to its extreme rarity.

## 2. Case Report

**Figure 1:**
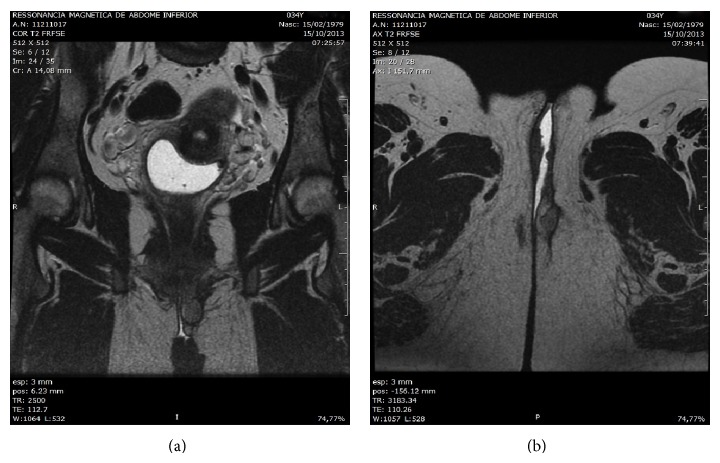
(a) Coronal and (b) axial MRI images with a 2.4 × 2 × 1.4 cm size soft tissue mass, in the left labia majora, and no pelvic and inguinal lymphadenopathy.

**Figure 2:**
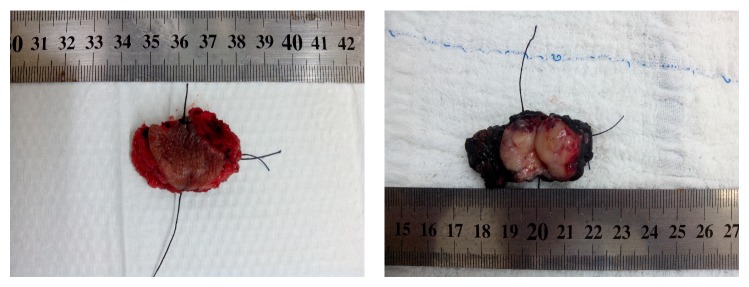
Excisional specimen of the vulvar proximal-type ES measuring 3.6 × 2.5 in size and 2.1 cm in depth.

**Figure 3:**
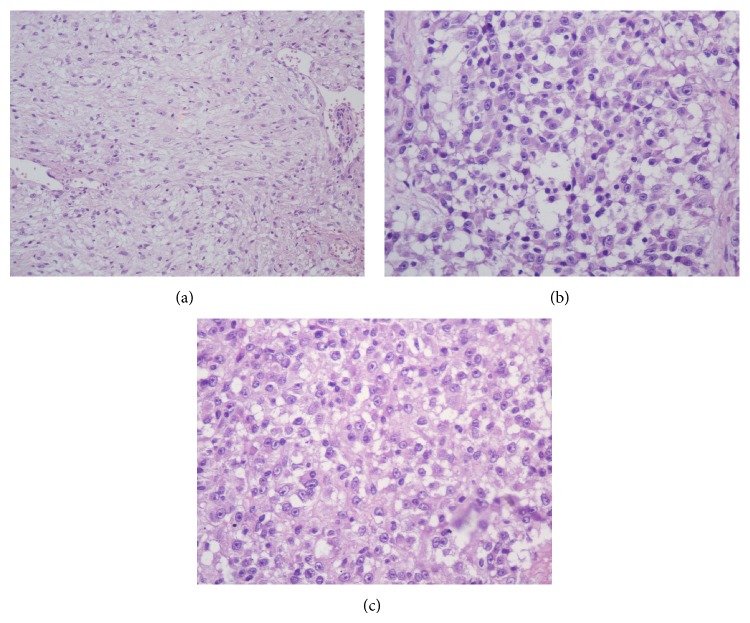
Histologic pictures. Paraffin sections * *with spindled or polygonal epithelioid cells in a myxoid stroma ((a) H&E, ×200; (b) H&E, ×400; (c) H&E, ×400).

**Figure 4:**
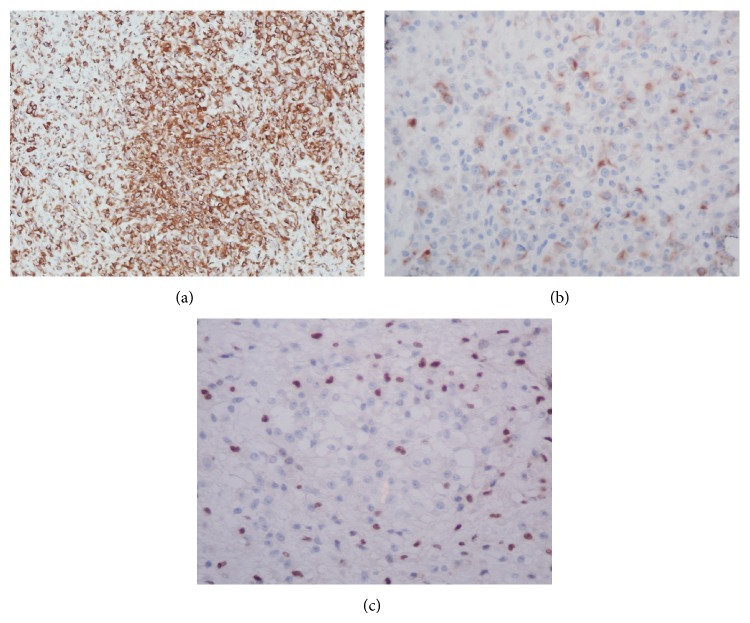
IHQ analysis. (a) The tumor cells presented diffuse positivity staining for vimentin and (b) focal positive staining for AE1/AE3. (c) The tumor cells showed loss of INI-1 protein expression.

A 34-year-old multiparous woman presented a palpable nodule in the posterior portion of the left labia majora, for 4 months. Excisional biopsy was performed. The initial pathology report suggested the diagnosis of epithelioid sarcoma, and therefore she was referred to Barretos Cancer Hospital. She had negative personal and family history for other malignancies. The clinical exam showed a nonulcerated subcutaneous nodule of 2 cm in the largest diameter in the left labia majora. The tumor was not tender, with a smooth surface and hard consistency. No other abnormalities were found. The pathology review described a 3.4 × 3 × 2 cm specimen, with positive margins. The diagnosis of epithelioid sarcoma, “proximal type,” with focal myxoid changes was made. Magnetic resonance imaging (MRI) of the pelvis showed a soft tissue tumor of 2.4 × 2 × 1.4 cm lesion in the left labia majora and no pelvic and inguinal lymphadenopathy (Figure 1). A chest and abdominal computerized tomography imaging (CT) did not show any evidence of metastasis. A wide local excision was performed, with 2 cm clean margins. Inguinal lymphadenectomy was not performed. The surgical specimen revealed a fragment measuring 3.6 × 2.5 in the largest diameters and 2.1 cm in depth with a mildly thickened epidermis. In the dermis, a well-circumscribed lobular nodule was observed, without ulceration (Figure 2). Microscopically, the tumor is composed of spindles or polygonal epithelioid cells in a myxoid stroma, without necrosis or vascular invasion. The cells had pleomorphic vesicular nuclei, prominent nucleolus, and eosinophilic cytoplasm (Figure 3). The surgical margins were free of tumor. Immunohistochemistry (IHQ) revealed strong positivity for the epithelial marker EMA (epithelial membrane antigen) and vimentin staining; focal positivity for cytokeratin AE1/AE3 and CD34; and it was negative for INI-1, S100, and CD31 (Figure 4), confirming the diagnosis. She underwent no postoperative adjuvant therapy. Eighteen months after operation, the patient is asymptomatic, with no evidence of disease.

## 3. Discussion

Vulvar myxoid epithelioid sarcoma is an exceptionally unusual tumor that represents a special entity due to its characteristic pathological appearance and clinical behavior. A vulvar site for a primary presentation of ES is rare and is frequently misdiagnosed as a benign lesion. Main differential diagnosis may include infectious granuloma, Bartholin cyst, fibroma, fibrous histiocytoma, fasciitis, lipoma, dermoid cyst, viral warts, or squamous cell carcinoma [4, 5]. The most common initial symptom of vulvar ES is a slowly growing, relatively painless mass in the labia majora [6]. Ulutin et al. reported a median time of 6 months for the diagnosis of vulvar sarcoma, resulting in a considerable delay in adequate treatment [7]. Sometimes, there may be up to two-year interval after the onset of initial symptoms, and the correct diagnosis is not made until the tumor is in an advanced stage [8]. Recent published studies showed genetic modification and loss of SMARCB1 protein expression in more than 80% of cases of ES [9–11]. Immunohistochemistry reveals cytoplasmic immunoreactivity for cytokeratin, vimentin, and EMA. CD 34, desmin, and HMB reactivity can be seen focally in some tumors. Usually, staining for S100 and staining for CD31 are negative. Immunostaining for INI1 can be used to confirm the diagnosis of ES as a complement to epithelial markers and CD34. In fact, absence of INI1 expression is rarely observed in other tumor types [9]. These immunohistochemical findings are useful for differentiating ES from the other malignant soft tissue tumors of epithelioid appearance, including synovial sarcoma, extrarenal malignant rhabdoid tumor, epithelioid malignant peripheral nerve sheath tumor, melanoma, rhabdomyosarcoma, and undifferentiated carcinoma [12]. Correct diagnosis can be challenging, and cases of benign and malignant myoepithelioma, extraskeletal myxoid chondrosarcoma, and epithelioid myxofibrosarcoma should be distinguished from myxoid ES [13]. In the described case, the IHQ studies supported the diagnosis of myxoid ES. Locoregional lymph node involvement, vascular invasion, tumor size larger than 2 cm, deep localization, presence of necrosis, and a high mitotic index in excess of 2 per 10 high power fields are poor prognosis risk factors [14, 15]. However, these prognostic factors have not been well established for vulvar ES due to its rare incidence [16]. Many authors prefer local wide excision with surgical margins of at least 2 cm to radical vulvectomy [17, 18]. The treatment of choice remains uncertain. Maintenance of fertility should be considered in patients with early lesions. Locoregional lymph node dissection for staging should only be considered in the presence of clinically suspicious or enlarged lymph nodes, because lymph node resection did not present a significant impact on local or distant relapse rate, although in some cases it may have a palliative intent [1]. The role of adjuvant radiotherapy and/or chemotherapy is not clear. Some reports advocate adjuvant radiotherapy in the presence of high-grade tumors or inadequate surgical margins [19]. Chemotherapy regimens for the treatment of recurrence were variable and achieved a modest benefit, with only one patient surviving more than a year after starting chemotherapy [20]. Many of the recurrences were described within the first 6 months of the initial treatment. A complete initial excision is a cornerstone, as inadequate margins are associated with an increased risk of local recurrence.

In this described case, the lesion was initially considered a benign condition, and an adequate treatment was delayed at least for 3 months. She underwent a biopsy of the vulvar mass as soon as the possibility of malignancy was raised. The local treatment of a vulvar ES includes a wide local excision of the lesion with adequate safety margins. Adjuvant therapy was not considered in the reported case because the lesion was completely resected, with a wide clean margin. Furthermore, there was no clinical or radiological evidence of regional/distant metastases. Within this short follow-up, there was no evidence of recurrence, but a long-term follow-up is necessary.

In conclusion, vulvar myxoid ES is a rare condition. It may appear as an extremely aggressive tumor or even as low-grade tumors. It is best treated by early diagnosis and wide local excision. Early diagnosis is not always possible, as it is frequently mistaken for benign lesions. The possibility of this rare tumor variant may be considered at the presence of a persistent and painless vulvar mass, to ensure correct diagnosis and treatment.
